# Transcriptome-based identification of antioxidative gene expression after fish oil supplementation in normo- and dyslipidemic men

**DOI:** 10.1186/1743-7075-9-45

**Published:** 2012-05-23

**Authors:** Simone Schmidt, Frank Stahl, Kai-Oliver Mutz, Thomas Scheper, Andreas Hahn, Jan Philipp Schuchardt

**Affiliations:** 1 Faculty of Natural Sciences at the Leibniz University of Hannover, Institute of Food Science and Human Nutrition, Am Kleinen Felde 30, 30167, Hannover, Germany; 2 Faculty of Natural Sciences at the Leibniz University of Hannover, Institute of Technical Chemistry, Callinstr 5, 30167, Hannover, Germany

**Keywords:** Omega-3 fatty acids, Dyslipidemia, Antioxidative defence, Glutathione, Matrix metalloproteinase, Catalase, Heme oxygenase, Cytochrome P450 enzyme, Oxylipines

## Abstract

**Background:**

The beneficial effects of omega-3 polyunsaturated fatty acids (n-3 PUFAs), especially in dyslipidemic subjects with a high risk of cardiovascular disease, are widely described in the literature. A lot of effects of n-3 PUFAs and their oxidized metabolites are triggered by regulating the expression of genes. Currently, it is uncertain if the administration of n-3 PUFAs results in different expression changes of genes related to antioxidative mechanisms in normo- and dyslipidemic subjects, which may partly explain their cardioprotective effects. The aim of this study was to investigate the effects of n-3 PUFA supplementation on expression changes of genes involved in oxidative processes.

**Methods:**

Ten normo- and ten dyslipidemic men were supplemented for twelve weeks with fish oil capsules, providing 1.14 g docosahexaenoic acid and 1.56 g eicosapentaenoic acid. Gene expression levels were determined by whole genome microarray analysis and quantitative real-time polymerase chain reaction (qRT-PCR).

**Results:**

Using microarrays, we discovered an increased expression of antioxidative enzymes and a decreased expression of pro-oxidative and tissue enzymes, such as cytochrome P450 enzymes and matrix metalloproteinases, in both normo- and dyslipidemic men. An up-regulation of catalase and heme oxigenase 2 in both normo- and dyslipidemic subjects and an up-regulation of cytochrome P450 enzyme 1A2 only in dyslipidemic subjects could be observed by qRT-PCR analysis.

**Conclusions:**

Supplementation of normo- and dyslipidemic subjects with n-3 PUFAs changed the expression of genes related to oxidative processes, which may suggest antioxidative and potential cardioprotective effects of n-3 PUFAs. Further studies combining genetic and metabolic endpoints are needed to verify the regulative effects of n-3 PUFAs in antioxidative gene expression to better understand their beneficial effects in health and disease prevention.

**Trial registration:**

ClinicalTrials.gov (ID: NCT01089231)

## Background

Cardiovascular disease (CVD) is the leading cause of morbidity and mortality in Europe, and frequently appears in subjects with disorders of lipid metabolism. Evidence of an association between dyslipidemia and increased oxidative stress [1,2], as well as between increased oxidative stress and the pathogenesis of CVD, are given by many studies [3-7]. These associations indicate that dyslipidemia increases oxidative stress, and thus promotes the pathogenesis of CVD.

Enhanced oxidative stress results from either an overproduction of reactive oxygen species (ROS) or a decreased antioxidative defence system. The most important ROS producers are nicotinamide adenine dinucleotide phosphate oxidase [8], xanthine oxidase [9], uncoupled endothelial nitric oxide synthase [10], and enzymes of the arachidonic acid (AA, 20:4n-6) metabolism and the mitochondria [11]. The consequences of an increased ROS production in CVD are vascular cell dysfunction [12], increased growth of the myocard, apoptosis [13], and cardiac remodelling via activation of matrix metalloproteinases (MMP) [14].

The human body possess enzymatic and non-enzymatic strategies to compensate oxidative damage and protect itself against such cytotoxic effects. Important antioxidative enzymes include catalase (CAT), superoxide dismutase (SOD), heme oxygenase (HMOX), and glutathione peroxidase (GPX). Non-enzymatic antioxidants, such as glutathione, ascorbate and α-tocopherol, are also important regulators of the oxidative status. In the last few decades, numerous observational and intervention studies have shown the beneficial effects of fish oil (FO) and its principal omega-3 polyunsaturated fatty acids (n-3 PUFAs), eicosapentaenoic acid (EPA, 20:5n-3) and docosahexaenoic acid (DHA, 22:6n-3), in the prevention of atherosclerosis and CVD [15-17]. Beyond the beneficial effects of n-3 PUFAs on the lipid profile [18-20], especially in subjects with hypertriglyceridemia [21-23], n-3 PUFAs appear to increase antioxidative capacity and thus reduce oxidative stress [24,25]. However, the effects of n-3 PUFAs on oxidative stress have not been studied in detail and some existing results are inconsistent. Investigations in patients with chronic renal failure showed reduced oxidative stress after n-3 PUFA supplementation [24]. Furthermore, in vitro studies with human aortic endothelial and HepG2 cells also determined reduced oxidative stress after n-3 PUFA treatment [25]. However, an indication of increased oxidative stress in healthy judo athletes after n-3 PUFA supplementation was observed [26]. The underlying molecular mechanisms by which EPA and DHA influence oxidative stress are not completely understood. Changes in expression levels of antioxidative genes in response to FO supplementation have not been investigated in dyslipidemic subjects so far. In regard to the fact that dyslipidemia increases oxidative stress and that dyslipidemic subjects possess a pro-oxidant status [27,28], we hypothesised that n-3 PUFAs show their potential antioxidative effects especially in dyslipidemic subjects. Therefore, this intervention trial aimed to investigate the expression changes of oxidative stress-related genes in normo- and dyslipidemic subjects after FO supplementation to gain information about the potential antioxidative effects of n-3 PUFAs.

## Methods

This study focused on changes in oxidative stress-related genes as part of a trial investigating the effects of FO supplementation on whole-genome gene expression profiles in normo- and dyslipidemic men. The randomized, controlled, parallel intervention study was conducted at the Institute of Food Science and Human Nutrition at the Leibniz University of Hannover in Germany, and was designed and performed according to the principles of the Good Clinical Practice Guidelines laid down in the Declaration of Helsinki. It was approved by the Freiburg International Ethical Commission and registered at ClinicalTrials.gov (ID: NCT01089231).

### Subjects

Normo- and dyslipidemic men were recruited by several advertisements and study placards in Hannover. The suitability of volunteers was checked in telephone interviews and by diet, lifestyle and disease questionnaires. Exclusion criteria comprised smoking; body mass index > 35; intake of any corticosteroids, lipid-lowering or anti-inflammatory drugs; diagnosed chronically cardiovascular or liver diseases; gastrointestinal disorders; blood coagulation disorders and intake of coagulation-inhibiting drugs; renal failure; periodic intake of laxatives; ingestion of supplements enriched with n-3 PUFAs, phytosterols, polyglucosamines, other lipid-binding ingredients or daily eating of fatty fish; allergy to fish or FO; and participation in another clinical study < 30 days before the start of the study or at the same time. Selected subjects were invited for a screening examination to collect fasting blood and determine serum lipid levels. Among these subjects, ten normolipidemic (total cholesterol (TC) < 200 mg/dl; low density lipoprotein cholesterol (LDL-C) < 130 mg/dl; triacyltriglyceride (TG) < 150 mg/dl) and ten dyslipidemic (TC > 200 mg/dl; LDL-C > 130 mg/dl; TG > 150 mg/ml) men, aged between 29 and 51 years, were enrolled in the study population. All participants included gave their written informed consent to take part in the study. The study protocol was approved by the Freiburger ethics committee.

### Study design

The normo- and dyslipidemic subjects each ingested six FO capsules per day for a period of twelve weeks. The daily n-3 PUFA intake for each subject via FO capsules was 2.7 g (1.14 g DHA and 1.56 g EPA). The subjects were instructed to take the capsules together with food, three in the morning and three in the evening, and to maintain their usual exercise and dietary habits throughout the intervention time. As an exception, on the first intervention day, all six capsules were ingested at the same time in the morning after a standardised breakfast. Additionally, participants completed a questionnaire to obtain information about changes in medication, diet (e.g. changes in weekly fish intake, preferred fish dishes or species, respectively) and lifestyle habits (e.g. physical activity), as well as the tolerability of the capsules.

### Determination of red blood cell membrane fatty acid composition

Fasting venous blood samples were collected into BD Vacutainer® Blood Collection Tubes (Becton Dickinson, Heidelberg, Germany). Red blood cell (RBC) membrane fatty acid (FA) composition, including the omega-3 index (EPA + DHA levels in RBC membranes), was analysed at baseline (t_0_) and after twelve weeks of supplementation with FO (t_12_), according to the omega-3 index methodology [29]. Accordingly, the RBCs were first transesterificated, resulting in a generation of FA methyl esters, followed by gas chromatography analysis using a GC2010 Gas Chromatograph (Shimadzu, Freising, Germany) equipped with a SP2560, 100 m column (Supelco, Bellefonte, PA) using hydrogen as the carrier gas. Identification of the FAs was enabled by comparison with a standard mixture of the FAs characteristic for RBCs. The results are presented as a percentage of the total FAs identified. The analytical coefficient of variation for EPA and DHA was 5%. Quality was assured according to DIN ISO 15189.

### Gene expression analyses

#### Sample collection

Fasting venous blood samples were collected in PAXgene Blood RNA Tubes (PreAnalytiX, Hombrechtikon, Switzerland) at baseline (t_0_), after one week (t_1_) and after twelve weeks (t_12_) of supplementation to analyse medium- and long-term effects of the FO supplementation on gene expression regulation. For short-term effects, venous blood samples were collected four hours (t_4h_) after the first intake of the capsules. The whole blood samples were collected and incubated for 24 hours in the PAXgene Blood RNA Tubes at room temperature. Whole blood samples were used for the RNA isolation and examination of gene expression because cell fractioning steps, such as lymphocyte isolation, could alter the gene expression pattern [30].

#### Total RNA isolation from human whole blood, RNA purification and sample pooling

The total RNA was isolated with the PAXgene Blood RNA Kit (PreAnalytiX, Hombrechtikon, Switzerland), according to the manufacturer’s recommended procedures. The RNA yield was quantified by Nanodrop ND-1000 spectrophotometer (peQLab Biotechnologie GmbH, Erlangen, Germany) measurement. The total RNA was purified with the Globin Clear Kit (Ambion, Applied Biosystems, Darmstadt, Germany), according to the manufacturer’s instructions. The reduction of highly abundant globin mRNA transcripts in whole blood samples is necessary to enable the detection of low-abundance transcripts [31]. The purified RNA was quantified again, and the quality was measured with an Agilent 2100 Bioanalyzer using RNA 6000 Nano Chips and a RNA 6000 Nano Kit (Agilent Technologies, Böblingen, Germany).

Equal amounts of purified RNA samples from each member of the respective group were pooled together. This was done for all different time points (t_0_, t_4h_, t_1_, and t_12_). Therefore, four pool samples were generated by this process for each group. The approach of sample pooling was chosen to reduce biological inter-individual variability, which is frequent due to variations in the relative proportions of specific blood cell subsets, gender, age, and disease state [32].

#### Microarray analysis (cDNA synthesis, hybridisation and data analysis)

First-strand cDNA synthesis and tyramide signal amplification (TSA) was performed using the Micromax TSA Labelling and Detection Kit (Perkin Elmer Life Sciences, Rodgau, Germany) with several protocol modifications. A total amount of 6 μg from every RNA pool, as well as random hexamer primer (Fermentas, St. Leon-Rot, Germany) and oligo(dT) primer (Roth, Karlsruhe, Germany), were used for the cDNA synthesis, which was facilitated by using Superskript III reverse transcriptase (Invitrogen, Karlsruhe, Germany). The incubation time of two hours was split into two one-hour incubations and additional Superskript III was added after the first hour. Each RNA pool was synthesized into two differently labelled cDNAs, fluorescein-labelled and biotin-labelled cDNA.

After labelling, the cDNA samples were purified with the QIAquick PCR Purification Kit (Qiagen, Hilden, Germany), according to the manufacturer’s instructions. Furthermore, the cDNA samples were first vacuum-dried and then resolved in hybridization buffer (4 x SSPE; 2.5 x Denhardt’s reagent; 30% formamid). After a final degradation step (3 minutes, 95°C), one-tenth of top-block (Sigma-Aldrich, Steinheim, Germany) was added. Equal amounts of biotin-labelled and fluorescein-labelled cDNA were hybridized simultaneously in oneexperiment to human whole genome OneArray™ Microarrays (Phalanx Biotech Group; Belmont, CA, USA). Hybridizations were carried out overnight at 42°C in hybridization chambers (Eppendorf AG, Hamburg, Germany). After hybridization, unbound and non-specific fixed cDNA was removed by stringent washing from the array. Specifically bound fluorescein- and biotin-labelled cDNA were sequentially detected with a series of conjugate reporter molecules according to the TSA process, ultimately with tyramide-Cy3 and tyramideCy5. Microarray experiments were performed for each study group in a loop design to prevent dye-dependent variety effects [33].

The array data were submitted to Gene Expression Omnibus [34], which supports minimum information about a microarray experiment [35]. The accession number of the submitted dataset is GSE34898. Genes that were detected as differentially expressed between baseline and time point t_4h_, t_1_ or t_12_ were subjected to pathway analysis using the Kyoto Encyclopaedia of Genes and Genomes (KEGG) database and GenMAPP [36].

#### Quantitative real-time polymerase chain reaction (qRT-PCR) and data analysis

In order to quantify the expression levels of selected genes, equal amounts of cDNA were synthesized using 2 μg of purified RNA and M-MLV reverse transcriptase (Promega, Mannheim, Germany), as well as random hexamer (Fermentas, St. Leon-Rot, Germany) and oligo(dT) primers (Carl Roth; Karlsruhe, Germany). Synthesized cDNA was diluted 1:20 with nuclease-free water and used for the qRT-PCR together with iQ SYBR Green Supermix (Bio-Rad Laboratories, Hercules, Ca, USA) and 5 pmol of both forward and reverse primers. The sequences for target and reference genes were retrieved from GenBank and applied primers were manually designed with the Primer-BLAST tool of the National Centre for Biotechnology Information, which is based on the program Primer3 [37]. The primer sequences used are listed in Table 1. Glyceraldehyde-3-phosphate dehydrogenase (GAPDH) and ribosomal protein S2 (RPS2) were identified as the most stable reference genes by the freely available algorithm geNorm version 3.5.

**Table 1:** Nucleotide sequences of primers for quantitative real-time polymerase chain reaction

	**Gene symbol**	**RefSeq_ID**	**Sequences (5′- > 3′)**
			forward CTGACACTCACCGCCATCGCC
	CAT	NM_001752.2	
			reverse TGTCCTGCATGCACATCGGGC
		NM_001127204.1	
			forward GCAGCAAGAACCACACCCAGCA
**Target genes**	HMOX2	NM_001127205.1	
			
		NM_001127206.1	
			reverse TGGGTGTTTTCTGCCCGGTCG
		NM_002134.3	
			forward AGCGCCGGTGTATCGGGGAAG
	CYP1A2	NM_000761.3	
			reverse TCAGTTGATGGAGAAGCGCAGCCG
			forward AAGGTGGTGAAGCAGGCGTCG
**Reference genes**	GAPDH	NM_002046.3	
			reverse AATGCCAGCCCCAGCGTCAAAG
			forward GCAACTTCGCCAAGGCCACCTT
	RPS2	NM_002952.3	
			reverse TGGGTCTTGACGAGGTGGTCAGT

### Statistics

Statistical analysis of blood lipids and RBC membrane FAs were processed with SPSS software version 20 (SPSS Inc., Chicago, IL, USA). The results are based on per protocol population, defined as subjects completing all visits not infringing the study protocol, and are presented as mean ± SD. Differences between baseline blood lipid values of both groups were tested by *t*-test. Differences of FAs in RBC membranes between t_0_ and t_12_ were tested within groups by paired *t*-test. Statistical significance was generally accepted at p ≤ 0.05. The arrays were scanned with a 4000 B scanner (Axon Instruments, Union City, CA, USA) and images were quantified using GenePixPro 6.0 software for the statistical analysis of the microarray data. The average pixel intensity within each spot was determined and a local background was computed for each spot. The net signal was determined by subtracting local background from the average intensity. Signals not consistently detectable (background corrected signal lower than twice background standard deviation) were excluded from further analysis. Following the primary analysis, data from different scans had to be summarized. The scans firstly had to be normalized by the sum of all corresponding spot-intensities due to different laser power and photomultiplier tube settings. Afterwards, data from different scans for each individual spot could be averaged by the mean. The mean of the data for differently labelled targets for each gene on two microarrays was taken. In order to deduce if expression of a gene is significantly different in the two samples, the preprocessed data was analyzed by hypothesis testing. It was assumed that the distribution of the preprocessed data was normal, and hence, a standard two-state pooled-variance *t*-test (1% and 5% probability of error) was applied in order to detect differentially expressed genes. The genes can be categorized into three groups using the calculated p-value of those t-tests [1: slightly significant regulation (p = 0.05); 2: significant regulation (p < 0.05); 3: highly significant regulation (p < 0.01)].

Statistical analysis of the expression ratios of genes, which were quantified by qRT-PCR, were calculated with the Gene Expression Macro tool (Bio-Rad), which is based on the algorithm of geNorm [38]. Firstly, normalization factors were calculated from the geometric mean of the reference genes GAPDH and RPS2. Furthermore, the baseline values of the normolipidemic group were defined as control values so that relative expression values could be calculated. Therefore, the baseline samples of the normolipidemic group are given a value of 1. Due to the lack of Gene Expression Macro tool to calculate statistics, differences between baseline and endpoint (t_12_) Ct values were tested by paired t-test using the statistical package R version 2.15.0.

## Results

### Subject characteristics

All 20 subjects (ten normolipidemic and ten dyslipidemic men) completed the study. No significant differences of the mean age and mean weight were observed between both study groups at baseline. The dyslipidemic subjects presented a 4.47 kg/m² higher BMI, higher TC and TG level, as well as a higher LDL-C/HDL-C quotient, than the normolipidemic subjects (Table 2). Subjects of the dyslipidemic group can be characterised as pre-obese (BMI 25–30), which is, among others, an underlying cause for dyslipidemia. The BMI was not changed by dietary intervention in either study groups (data not shown).

**Table 2:** **Subjects characteristics of normo- and dyslipidemic men at baseline (t**_**0**_**)**

**Parameters**	**Normolipidemic (n = 9)**	**Dyslipidemic (n = 7)**
Age [years]	36.56 ± 8.00	41.43 ± 6.63
Height [cm]	180.61 ± 6.56	180.28 ± 8.62
Weight [kg]	77.41 ± 15.44	91.84 ± 12.83
Body mass index (kg/m²]	23.66 ± 3.97 ^a^	28.13 ± 1.99 ^a^
Total cholesterol [mg/dl]	183.33 ± 13.88 ^a^	272.86 ± 67.1 ^a^
Triacylglycerol [mg/dl]	82.22 ± 37.42 ^a^	362.00 ± 284.62 ^a^
High density lipoprotein [mg/dl]	58.67 ± 10.92	45.86 ± 6.15
Low density lipoprotein cholesterol [mg/dl]	108.33 ± 13.54	146.60 ± 6.43
LDL-C/HDL-C quotient	1.90 ± 0.37 ^a^	3.10 ± 0.47 ^a^

a t0 values of normolipidemic subjects vs. t0 values of dyslipidemic subjects were tested by student’s *t*-test; p < 0.05.

### Fatty acid composition of RBC membranes and omega-3 index

No significant differences of EPA or DHA levels in RBC membranes or in the omega-3 index were observed between the study groups at baseline (Table 3). However, dyslipidemic subjects presented lower AA levels in RBC membranes than normolipidemic subjects at baseline. The percentage of EPA and DHA, and the omega-3 index in RBC membranes significantly increased within both study groups after twelve weeks of supplementation. Additionally, the normolipidemic group showed a significant decrease of the percentage of AA in RBC membranes.

**Table 3:** **Red blood cell membrane fatty acid composition of normo- and dyslipidemic men at baseline (t**_**0**_**) and after supplementation with fish oil over twelve weeks (t**_**12**_**)**

	**Normolipidemic (n = 9)**		**Dyslipidemic (n = 7)**	
Fatty acid [%]*	**t0**	**t12**	**t0**	**t12**
C20:4n-6 (AA)	16.04 ± 0.80 ^a^	13.09 ± 0.63 ^#^	12.71 ± 2.52 ^a^	11.56 ± 1.58
C20:5n-3 (EPA)	0.85 ± 0.20	3.85 ± 0.64 ^#^	1.02 ± 0.43	3.46 ± 0.68 ^#^
C22:6n-3 (DHA)	4.47 ± 0.67	6.92 ± 0.81 ^#^	3.85 ± 1.45	6.46 ± 0.77 ^#^
SFA	36.42 ± 1.46	39.24 ± 1.25 ^#^	36.74 ± 1.78	39.24 ± 1.74
MUFA	17.98 ± 1.64 ^a^	17.20 ± 1.02 ^b^	22.15 ± 3.44 ^a^	19.03 ± 1.18 ^b#^
PUFA	44.59 ± 2.37 ^a^	42.98 ± 1.16 ^#^	40.13 ± 2.90 ^a^	41.11 ± 1.71
n-3 PUFA	7.90 ± 0.94	14.71 ± 1.45 ^#^	7.33 ± 2.04	13.53 ± 1.49 ^#^
n-6 PUFA	36.68 ± 2.09 ^a^	28.27 ± 1.39 ^#^	32.80 ± 2.04 ^a^	27.58 ± 1.86 ^#^
Omega-3 index	5.32 ± 0.74	10.77 ± 1.10 ^#^	4.87 ± 1.83	9.92 ± 1.34 ^#^

* percentage of total fatty acids.

a t_0_ values of normolipidemic subjects vs. t_0_ values of dyslipidemic subjects were tested by student’s *t*-test; p < 0.05.

b t_12_ values of normolipidemic subjects vs. t_12_ values of dyslipidemic subjects were tested by student’s *t*-test; p < 0.05.

# t_0_ values vs. t_12_ values were tested by student’s paired *t*-test; p < 0.05.

### Regulation of gene expression by n-3 PUFA supplementation

It was necessary to exclude the RNA samples of one normo- and three dyslipidemic subjects from the microarray experiments and following data analysis due to several reasons: Low RNA yield (three subjects) and consumption of medication that led to exclusion (one subject). Therefore, RNA pools were generated and data was analysed from nine normolipidemic and seven dyslipidemic subjects for each investigation time point.

Microarray experiments showed that several genes related to different oxidative processes were regulated. These genes are listed with the respective regulation ratio for each time point in Table 4. Several enzymes of the glutathione metabolism are regulated after FO supplementation, particularly in dyslipidemic subjects. While genes related to the glutathione synthesis were similarly up- and down-regulated during the first two time points (t_4h_ and t_1_), these genes were mainly up-regulated after twelve weeks of FO supplementation. Two different glutathione transferases (GST) and glutathione reductase (GR) were up-regulated, whereas glutathione peroxidases were down-regulated in both normo- and dyslipidemic subjects. MMPs were down-regulated in both normolipidemic (MMP25) and dyslipidemic subjects (MMP2, MMP3) after twelve weeks of supplementation. Furthermore, cytochrome P450 (CYP) enzymes were mainly down-regulated after twelve weeks of supplementation, especially in dyslipidemic subjects. Additionally, some antioxidative enzymes, such SOD3, CAT and HMOX2, were up-regulated after twelve weeks of supplementation in dyslipidemic subjects. Moreover, pathway analysis discovered several regulated genes within stress-activated signalling pathways, such as the mitogen-activated protein kinase (MAPK) signalling pathway, the nuclear factor kappa b (NF_k_B) pathway and the oxidative stress athway (Additional file 1).

**Table 4:** Ratios of differentially expressed genes related to oxidative processes

**Regulated genes**	**Gene symbol**	**Entrez_ID**	**RefSeq_ID**	**Dyslipidemic**			**Normolipidemic**		
				**Ratio t4h : t0**	**Ratio t1 : t0**	**Ratio t12 : t0**	**Ratio t4h : t0**	**Ratio t1 : t0**	**Ratio t12 : t0**
**Gluthatione metabolism**									
Glutathione peroxidase 1	GPX1	2876	NM_000581.2	-	-	-	-	-	−2.48 ^3^
			NM_201397.1						
Glutathione S-transferase Mu 3	GSTM3	2947	NM_000849.4	−2.30 ^1^	4.12 ^2^	2.47 ^2^	-	-	-
Glutathione synthetase	GSS	2937	NM_000178.2	−7.97 ^1^	-	-	-	-	-
Phospholipid hydroperoxide glutathione peroxidase, mitochondrial	GPX4	2879	NM_001039848.1	-	−3.74 ^1^	-	-	-	-
			NM_001039847.1						
			NM_002085.3						
Glutathione S-transferase P	GSTP1	2950	NM_000852.3	-	−2.00 ^2^	-	-	-	-
Gamma-glutamyltransferase 5	GGTLA1	2687	NM_001099782.1	3.44 ^1^	14.90 ^1^	7.98 ^1^	-	-	-
			NM_001099781.1						
			NM_004121.2						
Glutathione peroxidase 3 (plasma)	GPX3	2878	NM_002084.3	-	-	−2.15 ^1^	-	-	-
Glutathione reductase	GSR	2936	NM_000637.2	-	-	2.38 ^3^	-	-	-
**Matrix metalloproteinases**									
Matrix metalloproteinase-25	MMP25	64386	NM_022468.4	-	-	-	2.14 ^2^	-	−2.30 ^1^
Metalloproteinase inhibitor 2	TIMP2	7077	NM_003255.4	-	−4.18 ^1^	−2.29 ^1^	-	-	-
stromelysin-1	MMP3	4314	NM_002422.3	-	-	−2.17 ^1^	-	-	-
72 kDa type IV collagenase	MMP2	4313	NM_001127891.1	-	−3.91 ^1^	−6.16 ^1^	-	-	-
			NM_004530.4						
**Cytochrom P450 enzymes**									
Cytochrome P450 1A2	CYP1A2	1544	NM_000761.3	-	-	-	-	-	−7.74 ^2^
Cytochrome P450 2A7	CYP2A7	1548	NM_000762.5	-	−2.46 ^1^	-		−2.42 ^1^	−2.70 ^1^
Cytochrome P450 4X1	CYP4X1	260293	NM_178033.1	-	−5.66 ^1^	−11.35 ^1^	-	-	-
Cytochrome P450 26A1	CYP26A1	1592	NM_057157.2	2.87 ^1^	-	-	-	-	-
			NM_000783.3						
Cytochrome P450 2B6	CYP2B6	1555	NM_000767.4	2.60 ^1^	-	-	-	-	-
Cytochrome P450 4F12	CYP4F12	66002	NM_023944.3	2.46 ^1^	-	-	-	-	-
Cholesterol side-chain cleavage enzyme, mitochondrial	CYP11A1	1583	NM_001099773.1	−2.30 ^1^	−2.78 ^1^	−2.79 ^1^	-	-	-
			NM_000781.2						
Cytochrome P450 26B1	CYP26B1	56603	NM_019885.2	−3.11 ^1^	-	-	-	-	-
Cytochrome P450 2C19	CYP2C19	1557	NM_000769.1	−7.48 ^1^	-	-	-	-	-
Steroid 17-alpha-hydroxylase/ 17,20 lyase	CYP17A1	1586	NM_000102.3	−7.96 ^1^	-	−4.19 ^1^	-	-	-
Cytochrome P450 2J2	CYP2J2	1573	NM_000775.2	-	-3.54 ^1^	-	-	-	-
Cytochrome P450 27C1	CYP27C1	339761	NM_001001665.3	-	−4.46 ^1^	−3.41 ^1^	-	-	-
Cytochrome P450 2A13	CYP2A13	1553	NM_000766.3	-	−4.49 ^1^	−8.90 ^1^			
**Others**									
Extracellular superoxide dismutase [Cu-Zn]	SOD3	6649	NM_003102.2	-	-	4.70 ^3^	-	-	2.91 ^3^
Catalase	CAT	847	NM_001752.2	-	13.15 ^1^	8.90 ^1^	-	-	-
Heme oxygenase 1	HMOX1	3162	NM_002133.1	-	−8.57 ^1^	−17.52 ^1^	-	-	-
Heme oxygenase 2	HMOX2	3163	NM_001127204.1	-	−11.44 ^1^	7.84 ^1^	-	-	-
			NM_001127205.1						
			NM_001127206.1						
			NM_002134.3						
Epoxide hydrolase 1	EPHX1	2052	NM_000120.3	-	-	3.14 ^2^	−2.24 ^2^	-	2.22 ^2^
			NM_001136018.2						
Arachidonate 5-lipoxygenase- activating protein	ALOX5AP	241	NM_001629.2	-	-	6.97 ^1^	-	-	-
Nitric oxide synthase, endothelial	NOS3	4846	NM_000603.4	-	−4.22 ^1^	−2.55 ^1^	-	-	-
Nitric oxide synthase, inducible	NOS2	4843	NM_000625.4	-	−8.11 ^1^	−4.77 ^1^	-	-	-
Nitric oxide synthase-interacting protein	NOSIP	51070	NM_015953.3	-	-	-	-	-	3.64 ^1^
NADPH oxidase 1	NOX1	27035	NM_013955.2	-	-	2.06 ^1^	-	-	-
			NM_007052.4						

Expression ratios were displayed for genes which were differentially expressed after four hours (t4h), one week (t1) and twelve weeks (t12) of fish oil supplementation in normolipidemic and dyslipidemic men.

- no regulation.

^1^ slightly significant regulation; p = 0.05.

^2^ significant regulation; p < 0.05.

^3^ highly significant regulation; p < 0.01.

**Figure 1:**
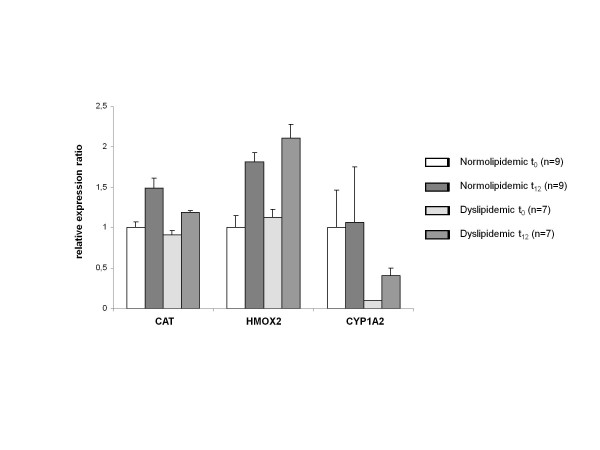
**Transcript levels of target genes in normo- and dyslipidemic men.** Transcript levels of catalase (CAT), heme oxygenase 2 (HMOX2) and cytochrome P450 enzyme 1 A2 (CYP1A2) was determined by qRT-PCR in normo- and dyslipidemic men before (t_0_) and after twelve weeks (t_12_) of fish oil supplementation. Pooled group samples were used in triplicates. Triplicates were averaged and corrected by two reference genes, glyceraldehyde-3-phosphate dehydrogenase (GAPDH) and ribosomal proteine S2 (RPS2). Corrected expressions were compared with baseline gene expression of normolipidemic subjects and relative expression changes are displayed. Differences between baseline and endpoint (t_12_) Ct values were tested by a paired t-test and differences between groups at each time point were tested by unpaired t-test.

Several genes were selected for analyses of gene expression ratios by qRT-PCR, including the two antioxidative enzymes, CAT and HMOX2, and CYP1A2 (Figure 1), a member of the CYP family known to be involved in epoxidation of EPA and DHA [39]. The expression of HMOX2 was significantly up-regulated after FO supplementation over a period of twelve weeks in both normo- and dyslipidemic subjects (p=0.02 and p=0.04). The expression of CAT was also up-regulated, but reached significance only in normolipidemic subjects (p=0.002). The expression of CYP1A2 was significantly up-regulated only in dyslipidemic subjects (p=0.04). The qRT-PCR results mainly confirm the microarray results observed, whereupon differences in the strength of expression occur.

## Discussion

To the best of our knowledge, this is the first intervention study disclosing gene expression changes in normo- and dyslipidemic subjects after FO supplementation. We identified several genes involved in oxidative processes, which were regulated by FO. The expression of antioxidative enzymes was up-regulated particularly in dyslipidemic subjects, while the expression of pro-oxidative or tissue damage-related enzymes was down-regulated. We suggest that n-3 PUFAs may have an antioxidative potential.

Antioxidative effects could be facilitated by either a reduced production of ROS or an increased production of antioxidative enzymes. Several human studies and in vitro experiments showed reduced superoxide or ROS production by monocytes and neutrophils after n-3 PUFA administration [40-42]. Additionally, negative correlations between ROS production and n-3 PUFA membrane content in healthy [40] and dyslipidemic subjects [41] were observed. On the other hand, positive correlations between the n-3 PUFA membrane content and the activity of antioxidative enzymes could be investigated in fibroblasts cell cultures [43] and in type 2 diabetes patients [44]. In the present study, the supplementation of normo- and dyslipidemic subjects with FO resulted in decreasing AA levels in RBC membranes in favour of EPA and DHA, whose levels increased considerably. Accordingly, the increase of EPA and DHA levels observed in RBC membranes together with an increased expression ratio of the antioxidative enzymes CAT and HMOX2 are in agreement with the findings of Benito [43] and Smaoui [44]. Moreover, the replacement of AA, which is an important ROS producer, in biological membranes may partly explain the antioxidative properties of n-3 PUFAs. On the other hand, the incorporation of EPA and DHA in RBC membranes in response to long-term n-3 PUFA administration results in increased induced lipid peroxidation [45]. In this context, an activation of antioxidative gene expression in response to n-3 PUFA supplementation might be a reaction of the defence system to lower lipid peroxidation. Complementary analysis of oxidative damage or oxidative stress markers combined with expression changes of anti- and pro-oxidant genes should be used to indentify global antioxidative effects.

Nevertheless, an increased expression of HMOX2 and CAT in normo- and dyslipidemic subjects may indicate some antioxidative effects of n-3 PUFAs. To our knowledge, this is the first study at all showing a regulation of HMOX2 expression after n-3 PUFA supplementation in humans. HMOXs are antioxidative enzymes which catabolise heme to biliverdin and carbon monoxide. The two existing HMOXs 1 and 2 differ in their activity. HMOX2 is constitutively expressed, whereas HMOX1 is inducible, e.g. by cellular stress [46]. HMOX2 was identified as part of the large-conductance calcium and voltage-activated potassium (BK(Ca)) channel complex and could enhance its activity, while knockdown of HMOX2 expression reduced channel activity [47]. BK(Ca) channels could influence the cell membrane potential and, therefore, play an important role in many physiological functions, including oxygen-sensing, neuronal excitability, vascular tone regulation, and neurotransmitter release [48,49]. However, a possible clinical relevance of an increased HMOX2 expression after FO supplementation has to be clarified in further studies.

CAT is an effective antioxidative enzyme [50] known to compensate H_2_O_2_[51,52], e.g. in the centre of inflammation [53,54]. In this study, expression ratios of the microarray experiments showed an increased expression of CAT in dyslipidemic subjects, whereas qRT-PCR showed an increased expression in both study groups, reaching statistical significance only in normolipidemic subjects. These differences are also known from several other gene expression studies and are mainly explained by the greater sensitivity of the qRT-PCR [55,56]. The increased expression of CAT in normolipidemic subjects is in contrast to studies with healthy volunteers, which mostly showed no effects on CAT activity after FO supplementation [57,58]. Results from animal studies, however, indicated an increased CAT activity after treatment with n-3 PUFA [59,60]. Human studies analysing the effects of n-3 PUFAs on the activity or expression of CAT in dyslipidemic subjects are very limited. In accordance with our results, Bouzidi and coworkers [24] reported an increased CAT activity in patients with dyslipidemia and chronic renal failure after n-3 PUFA supplementation, assuming a greater protection against oxidative stress and prevention of vascular complications. Similarly, an animal study with hypercholesterolemic rats also observed increased CAT activity after DHA feeding. Taken together, these findings suggest that longterm supplementation with n-3 PUFAs results in an enhanced capacity to detoxify H_2_O_2_ and might induce adaptive changes in the antioxidative defence system [61].

Glutathione is an important antioxidant which could be readily oxidized non-enzymatically to glutathione disulfide [62]. Most studies analysing the effects of n-3 PUFA supplementation on the activity of glutathione metabolism related enzymes, such as GPX, gamma-glutamylcysteine synthetase (gamma-GCL), GST, and GR, in healthy and dyslipidemic subjects showed increased activities of these enzymes [63-65]. In our study, the expression of GST and GR was increased in dyslipidemic subjects, while the expression of GPX was decreased in both normo- and dyslipidemic subjects. The increased expression of GST and GR is an indication of an increased glutathione synthesis and, therefore, an increased antioxidative defence status. GPX is recognized as an antioxidative enzyme which oxidizes glutathione to reduce and detoxify H_2_O_2_. Consequently, this enzyme is required when H_2_O_2_ levels rise in phases of oxidative stress [66,67]. Therefore, a decreased expression of GPX - observed in this study - could be an indicator of decreased oxidative stress. However, the results in the literature are inconsistent. Mabile and co-workers could not observe a change in the GPX activity in healthy and hypertriglyceridemic subjects [68], while other studies reported a stimulated GPX activity after n-3 PUFA supplementation in healthy [63] and hyperlipidemic subjects [64]. Furthermore, it was shown that DHA increased the activity of GST, gammaGCL and GR, as well as the mRNA expression of gamma-GCL and GR [65], in human fibroblasts, which is in agreement with our results.

CYP enzymes catalyze the oxidation of xenobiotic substances, such as pharmaceuticals, but also metabolize many endogenous substances, such as lipids and steroidal hormones. Besides cyclooxygenases and lipoxygenases, CYPs are also involved in the metabolism of PUFAs to form numerous different oxidized FA metabolites, also named oxylipines. The CYP isoforms of families 1 to 3 are mainly epoxygenases, and CYP isoforms from family 4 are mainly ω-hydroxylases [69]. In this study, several CYPs, mostly isoforms of family 2, were regulated after FO supplementation. The oxidation of EPA and DHA by epoxygenases could produce epoxy-derivates [70] and highly anti-inflammatory resolvins and protectins [71].

Generated EPA and DHA epoxides are effective dilators of coronary arterioles, facilitated by the activation of calcium-activated potassium channels [72,73]. The qRT-PCR showed that CYP1A2, which is one of the most efficient CYPs for the epoxidation of EPA and DHA in human liver microsomes [39], was up-regulated in dyslipidemic subjects after FO supplementation, suggesting the formation of specific EPA and DHA epoxides. Expression ratios of the microarray experiments showed decreased expression of CYP1A2 in normolipidemic subjects, which was, however, not confirmed by qRT-PCR. According to qRT-PCR experiments, the expression of CYP1A2 in normolipidemic subjects was not affected by FO treatment. Both results are in contrast to microarray experiments, where CYP1A2 was unregulated in dyslipidemic subjects and down-regulated in normolipidemic subjects. In view of the higher accuracy of qRT-PCR, it is suggested that the microarray result for CYP1A2 was false positive for normolipidemic subjects, while the microarray technique was insensitive to analyse the up-regulation of CYP1A2 in dyslipidemic subjects, which was generally much weaker. Interestingly, human liver microsomes, which were incubated with EPA and DHA (200 μM) showed a decreased CYP1A2 activity [74]. Although the results are contradictory, it has been repeatedly shown that n-3 PUFAs could induce the expression or activity of CYP enzymes, resulting in the formation of EPA and DHA metabolites [39,69,70,74,75]. The complex formation of n-3 PUFA metabolites by CYPs has not been investigated systematically so far; however, it is likely that the formation of these metabolites may explain numerous of the anti-inflammatory and cardioprotective effects of n-3 PUFAs [76].

MMPs are zinc-based proteases and could cleave macromolecules of the extra cellular matrix (ECM), e.g. collagens, as well as non-ECM molecules, such as growth factors, cytokines and their receptors [77]. ROS could induce the activity of MMPs [78], which could result in tissue remodelling processes [79] and promote the pathogenesis of several CVDs [80,81]. In this study, MMP2 and MMP3 in dyslipidemic subjects and MMP25 in normolipidemic subjects were down-regulated after FO supplementation. In accordance with our results, several other authors have shown decreased MMP2 and/or MMP9 expression or activity by n-3 PUFA in dyslipidemic subjects [82] and human cell cultures [83,84]. However, no changes in MMP9 activity were detected after FO supplementation in patients with coronary heart disease [85]. Similarly, another study observed a slight increase of the MMP2 activity in hypertriglyceridemic men after FO supplementation [86]. Further studies are needed to clarify these discrepancies and the function of n-3 PUFAs in the regulation of MMPs with regard to potential cardioprotective effects.

### Strengths and limitations

The methodological approach of this study was carefully elaborated. The use of whole blood for RNA isolation is advantageous in view of the easy sample collection and the prevention of altered gene expression patterns, which is a potential risk of cell fractionation steps [30]. In addition, the pooling of RNA samples reduces inter-individual variation, enabling one to focus on the effects of FO supplementation on the population level in contrast to an individual level [87]. However, the approach of sample pooling provides several limitations, primarily the reduction of statistical power. Finally, oxidative damage and oxidative stress markers were not analysed in this study, which complicates the evaluation of the antioxidative effects.

## Conclusions

In conclusion, this study showed indications of the antioxidative potential of n-3 PUFAs, especially in dyslipidemic subjects. FO supplementation resulted in an increased expression of glutathione synthesis-related genes, an up-regulation of antioxidative enzymes, such as CAT and HMOX2, and a reduced expression of MMPs and several CYPs. Interestingly, CYP1A2 was up-regulated in dyslipidemic subjects, suggesting an increased formation of n-3 epoxides. Taken together, these results indicate that n-3 PUFAs may have numerous different possibilities to reduce oxidative stress. It appears that n-3 PUFAs not only up-regulates antioxidative enzymes, but rather induces a specific interplay of differential regulations to generate an optimal balance of the oxidative status. Although the molecular mechanisms by which n-3 PUFAs mediate potential antioxidative effects cannot be clarified here, we hypothesise an involvement of PPARs. In vitro studies with human hepatocytes and pancreatic ß-cells have demonstrated an activation of PPAR-α or -γ by n-3 PUFAs, which resulted in an increased expression of CAT, as well as antioxidative effects [88,89]. Beside CAT, HMOX-1 has also been demonstrated as a target gene of PPAR [90]. Moreover, an increased expression of antioxidative genes could result in reduced oxidative stress, which further influences stress-activated pathways (MAPK and NF_k_B pathways), as well as other stress-related genes such as MMPs. However, studies analysing the expression of antioxidative enzymes, oxidative signalling processes and metabolic outcomes are needed to clarify the exact role of n-3 PUFAs within the antioxidative defence system.

### Availability of supporting data

The data sets supporting the results of this article are included within the article.

## Abbreviations

AA, Arachidonic acid; BK(Ca), Channel large-conductance calcium and voltage-activated potassium channel; CAT, Catalase; CVD, Cardiovascular disease; CYP, Cytochrome P 450; DHA, Docosahexaenoic acid; ECM, Extra cellular matrix; EPA, Eicosapentaenoic acid; FA, Fatty acid; FO, Fish oil; gamma-GCL, gamma-glutamylcysteine synthetase; GAPDH, Glyceraldehyde-3-phosphate dehydrogenase; GPX, Glutathione peroxidase; GR, Glutathione reductase; GST, Glutathione-S-transferase; H2O2, Hydrogenperoxide; HMOX, Heme oxygenase; LDL-C, Low density lipoprotein; MAPK, Mitogen-activated protein kinase; MMP, Matrix metalloproteinase; n-3 PUFA, Omega-3 polyunsaturated fatty acid; NFkB, Nuclear factor kappa b; qRT-PCR, Quantitative real-time polymerase chain reaction; RBC, Red blood cell; ROS, Reactive oxygen species; RPS2, Ribosomal protein S2; SOD, Superoxide dismutase; t, time point; TC, Total cholesterol; TG, Triacylglyceride; TSA, Tyramide signal amplification.

## Authors’ contributions

SS was involved in the study, experimental design, data analysis, interpretation, and manuscript writing. The study was mainly performed by SS. FS was involved in the experimental design and informed advice. KOM was involved in the experimental design, data analysis and manuscript editing. JPS was involved in study design, data interpretation and manuscript writing. The group leader of the Institute of Technical Chemistry, TS, was involved in the study design and manuscript editing. The group leader of the Institute of Food Science and Human Nutrition, AH, was involved in the study design and manuscript editing. Both JPS and AH were coordinators of the study. All authors have read and approved the final manuscript.

## Supplementary Material

Additional file 1 Regulated genes within stress-activated pathways. Genes, which were regulated after four hours (t_4h_), one week (t_1_) and twelve weeks (t_12_) of fish oil supplementation in normolipidemic and dyslipidemic men were submitted to pathway analyses according to the KEGG database as well as performed with GenMAPP. Expression ratios of regulated genes within mitogen-activated protein kinase (MAPK) signalling pathway, nuclear factor kappa b (NF_k_B) pathway and oxidative stress pathway were displayed. Click here for file
