# Supplementary material for: Transcriptome-based identification of antioxidative gene expression after fish oil supplementation in normo- and dyslipidemic men
Source: Nutr Metab (Lond). 2012 May 23;9:45. doi: 10.1186/1743-7075-9-45 (PMC3408332; doi:10.1186/1743-7075-9-45)
Supplement: Additional file 1 — Regulated genes within stress-activated pathways. Genes, which were regulated after four hours (t4h), one week (t1) and twelve weeks (t12) of fish oil supplementation in normolipidemic and dyslipidemic men were submitted to pathway analyses according to the KEGG database as well as performed with GenMAPP. Expression ratios of regulated genes within mitogen-activated protein kinase (MAPK) signalling pathway, nuclear factor kappa b (NFkB) pathway and oxidative stress pathway were displayed. [file 1743-7075-9-45-S1.pdf]

Additional file 1

Regulated genes within stress-activated pathways

Genes, which were regulated after four hours (t_{4h}), one week (t_1) and twelve weeks (t_{12}) of fish oil supplementation in normolipidemic and dyslipidemic men were submitted to pathway analyses according to the KEGG database as well as performed with GenMAPP. Expression ratios of regulated genes within mitogen-activated protein kinase (MAPK) signalling pathway, nuclear factor kappa b (NF_kB) pathway and oxidative stress pathway were displayed.

Gene name	Gene symbol	Entrez_ID	RefSeq_ID	Dyslipidemic			Normolipidemic		
				Ratio $t_{4h} : t_0$	Ratio $_{t1} : t_0$	Ratio $t_{12} : t_0$	Ratio $t_{4h} : t_0$	Ratio $_{t1} : t_0$	Ratio $t_{12} : t_0$
**Mitogen-activated protein kinase (MAPK) signaling pathway**									
Arrestin beta-1	ARRB1	408	NM_004041.3<br>NM_020251.2	-	-	8.60[1]	-	-	-2.12[2]
Arrestin beta-2	ARRB2	409	NM_199004.1<br>NM_004313.3	2.13[2]	3.86[3]	2.58[2]	-	-2.37[2]	-
V-raf murine sarcoma viral oncogene homolog B1	BRAF	673	NM_004333.4	-2.16[1]	-2.93[1]	-3.43[1]	-	-	-
Monocyte differentiation antigen CD14 Precursor	CD14	929	NM_000591.2<br>NM_001040021.1	4.82[3]	-	2.88[2]	-	-	-
Dual specificity phosphatase Cdc25B	CDC25B	994	NM_021873.2<br>NM_021872.2<br>NM_004358.3	-2.07[1]	-2.66[1]	-	-	-	2.83[1]
Cell division cycle 42	CDC42	998	NM_001791.3<br>NM_001039802.1	-	-	-	-	-	14.42[1]
Dual specificity protein phosphatase 1	DUSP1	1843	NM_004417.2	-2.071	-9.67[1]	-	-2.39[2]	-2.53[1]	-
Dual specificity protein phosphatase 8	DUSP8	1850	NM_004420.2	-	-4.37[1]	-3.09[1]	-	-	-
ETS domain-containing protein Elk-4	ELK4	2005	NM_021795.2<br>NM_001973.2	-	-	2.38[2]	-	-	-
Fibroblast growth factor 3	FGF3	2248	NM_005247.2	-	-4.42[1]	-16.68[1]	-	-	-
Fibroblast growth factor receptor 4 Precursor	FGFR4	2264	NM_022963.2<br>NM_213647.1<br>NM_002011.3	-	-	2.08[1]	-	-	-
MAP kinase-interacting serine/threonine-protein kinase 2	MKNK2	2872	NM_017572.3<br>NM_199054.2	-	-	2.25[2]	-	-	-2.04[1]
Growth factor receptor-bound protein 2	GRB2	2885	NM_203506.2<br>NM_002086.4	-	-2.94[2]	-	-	2.36[3]	-
Heat shock 70 kDa protein 6	HSPA6	3310	NM_002155.3	-	-	-	2.41[1]	-	-
Heat shock 70kDa protein 8	HSPA8	3312	NM_153201.1<br>NM_006597.3	-	-3.04[1]	-	-	2.63[1]	4.80[1]
Heat shock protein beta-1	HSPB1	3315	NM_001540.3	-	-2.29[1]	2.29[2]	-	-	-
Transcription factor jun-D	JUND	3727	NM_005354.4	-	2.14[1]	8.66[1]	-	-	-
GTPase KRas Precursor	KRAS	3845	NM_033360.2<br>NM_004985.3	-	-2.86[1]	-3.97[1]	-	-	-

Gene name	Gene symbol	Entrez_ID	RefSeq_ID	Dyslipidemic			Normolipidemic		
				Ratio $t_{4h} : t_0$	Ratio $t_1 : t_0$	Ratio $t_{12} : t_0$	Ratio $t_{4h} : t_0$	Ratio $t_1 : t_0$	Ratio $t_{12} : t_0$
**Mitogen-activated protein kinase (MAPK) signaling pathway [continuation]**									
Protein max	MAX	4149	NM_145113.1<br>NM_145112.1<br>NM_002382.3	5.49[1]	-	5.05[1]	-	-2.62[1]	-
Mitogen-activated protein kinase kinase kinase 3	MAP3K3	4215	NM_203351.1<br>NM_002401.3	-	-3.53[1]	-5.15[1]	-	-	-
Mitogen-activated protein kinase kinase kinase 5	MAP3K5	4217	NM_005923.3	-	-	-3.65[1]	-	-	-
Mitogen-activated protein kinase kinase kinase 11	MAP3K11	4296	NM_002419.3	-	2.71[1]	-	-	-	-
High affinity nerve growth factor receptor Precursor	NTRK1	4914	NM_001012331.1<br>NM_002529.3<br>NM_001007792.1	4.93[1]	-	-	-	-	-
cAMP-dependent protein kinase catalytic subunit alpha	PRKACA	5566	NM_207518.1<br>NM_002730.3	-	-5.55[1]	-	-	-	-
Protein kinase C alpha type	PRKCA	5578	NM_002737.2	-4.94[1]	-	-2.60[1]	-	-	-
Protein kinase C beta type	PRKCB	5579	NM_002738.6	-	12.47[1]	23.65[1]	-	-	-
Mitogen-activated protein kinase 8	MAPK8	5599	NM_139049.1<br>NM_139047.1<br>NM_139046.1<br>NM_002750.2	-	-2.90[1]	-2.52[1]	-	-	-
Dual specificity mitogen-activated protein kinase kinase 3	MAP2K3	5606	NM_002756.3<br>NM_145109.2	-	-6.68[1]	-	-2.34[2]	-	-
Tyrosine-protein phosphatase non-receptor type 7	PTPN7	5778	NM_080588.1<br>NM_002832.2	3.51[1]	-	-	-	-	-
Ras-related C3 botulinum toxin substrate 2	RAC2	5880	NM_002872.3	2.01[1]	-	-	-2.58[2]	-2.76[1]	-
Mitogen-activated protein kinase kinase kinase kinase 2	MAP4K2	5871	NM_004579.2	-	-8.82[1]	-	-	-	2.78[1]
Ras GTPase-activating protein 1	RASA1	5921	NM_002890.1<br>NM_022650.1	-	14.46[1]	16.67[1]	-	-	-
Ribosomal protein S6 kinase alpha-1	RPS6KA1	6195	NM_002953.3<br>NM_001006665.1	-	-5.52[1]	2.99[2]	-	-	2.32[3]
Dual specificity mitogen-activated protein kinase kinase 4	MAP2K4	6416	NM_003010.2	-2.35[1]	-	-2.72[1]	-	-	-
TGF-beta receptor type-2 Precursor	TGFBR2	7048	NM_003242.5<br>NM_001024847.2	8.64[1]	-	5.95[1]	-3.10[1]	-2.36[1]	-
Interleukin-1 receptor type II	IL1R2	7850	NM_173343.1<br>NM_004633.3	-	-5.50[1]	-	-	-	-

Gene name	Gene symbol	Entrez_ID	RefSeq_ID	Dyslipidemic			Normolipidemic		
				Ratio t_{4h} : t_0	Ratio t_1 : t_0	Ratio t_{12} : t_0	Ratio t_{4h} : t_0	Ratio t_1 : t_0	Ratio t_{12} : t_0
**Mitogen-activated protein kinase (MAPK) signaling pathway [continuation]**									
MAP kinase-activated protein kinase 5	MAPKAPK5	8550	NM_139078.1<br>NM_003668.2	-	-9.40[1]	-6.52[1]	-	-	-
Tumor necrosis factor receptor superfamily member 1A	TNFRSF1A	7132	NM_001065.2	-	-10.73[1]	-	-2.74[2]	-3.62[1]	-
Ribosomal protein S6 kinase alpha-4	RPS6KA4	8986	NM_003942.2<br>NM_001006944.1	-4.00[1]	-4.65[1]	-3.42[1]	-	-	-
Mitogen-activated protein kinase kinase kinase 6	MAP3K6	9064	NM_004672.3	-	-	-	-	-	2.34[1]
Serine/threonine-protein kinase TAO2	TAOK2	9344	NM_016151.2	-	-	3.04[1]	-	2.16[1]	-
Mitogen-activated protein kinase kinase kinase kinase 4	MAP4K4	9448	NM_145687.2<br>NM_004834.3<br>NM_145686.2	-	-	-6.73[2]	-	-	-
Rap guanine nucleotide exchange factor 2	RAPGEF2	9693	NM_014247.2	-	-2.73[1]	-	-	-	-
Mitogen-activated protein kinase kinase kinase 7-interacting protein 1	TAB1	10454	NM_006116.2	4.41[1]	16.23[1]	6.79[1]	-	-	-
Calcium-binding protein p22	CHP	11261	NM_007236.4	5.03[3]	-	-2.58[1]	-	-	-
Mitogen-activated protein kinase kinase kinase 7-interacting protein 2	TAB2	23118	NM_015093.3	-	-3.91[1]	-9.24[1]	-	-	-
Voltage-dependent calcium channel gamma-5 subunit	CACNG5	27091	NM_014404.1	-	-5.04[1]	-9.92[1]	-	-	-
Group IIE secretory phospholipase A2	PLA2G2E	30814	NM_014589.1	-	-	2.51[1]	-2.01[1]	-	-
Voltage-dependent calcium channel gamma-6 subunit	CACNG6	59285	NM_031897.2<br>NM_145815.1<br>NM_145814.1	-	-	-	-	2.92[1]	5.89[1]
Calcineurin B homologous protein 2	CHP2	63928	NM_022097.2	-	-5.75[1]	-5.31[1]	-	-	-
Protein kinase C eta type	PRKCH	5583	NM_006255.3	-	-2.91[1]	-2.36[1]	-	-	-
Mitogen-activated protein kinase 4	MAPK4	5596	NM_002747.3	-	-13.56[1]	-2.92[1]	-	-	-
Member of RAS oncogene family	RAPIB	5908	NM_001010942.2<br>NM_001251917.1<br>NM_001251918.1<br>NM_001251921.1<br>NM_001251922.1<br>NM_015646.5	-	4.15[1]	2.05[1]	-	-	-

Gene name	Gene symbol	Entrez_ID	RefSeq_ID	Dyslipidemic			Normolipidemic		
				Ratio t_{4h} : t_0	Ratio $_{t1}$: t_0	Ratio t_{12} : t_0	Ratio t_{4h} : t_0	Ratio $_{t1}$: t_0	Ratio t_{12} : t_0
**Mitogen-activated protein kinase (MAPK) signaling pathway [continuation]**									
Dual specificity mitogen-activated protein kinase kinase 6	MAP2K6	5608	NM_002758.3	-	-3.25[1]	-2.89[1]	-	-	-
Protein kinase C zeta type	PRKCZ	5590	NM_001033581.1<br>NM_001033582.1<br>NM_001242874.1<br>NM_002744.4	-2.65[1]	-	-	-	-	-
Caspase-9	CASP9	842	NM_032996.1<br>NM_001229.2	3.69[1]	-	-	-	-	-
Caspase-1	CASP1	834	NM_033293.2<br>NM_033292.2<br>NM_001223.3	2.12[1]	-	-	-	-	-
ECSIT homolog (Drosophila)	ECSIT	51295	NM_001142464.2<br>NM_001142465.2<br>NM_001243204.1<br>NM_016581.4	-	8.23[1]	8.72[1]	-	-	-
Receptor-interacting serine/threonine-protein kinase 1	RIPK1	8737	NM_003804.3	-	3.45[1]	2.20[2]	-	-	-
**Nuclear factor kappa b pathway**									
Alkaline phosphatase, tissue-nonspecific isozyme	ALPL	249	NM_000478.4<br>NM_001127501.2<br>NM_001177520.1	-	-3.47[1]	2.03[2]	-	2.42[1]	2.81[1]
Tumor necrosis factor receptor superfamily member 8	TNFRSF8	943	NM_152942.2<br>NM_001243.3	7.79[1]	-	-	-	-	-
Tumor necrosis factor receptor superfamily member 1A	TNFRSF1A	7132	NM_001065.2	-	-10.73[1]	-	-2.74[2]	-3.62[1]	-
Protein kinase C zeta type	PRKCZ	5590	NM_001033581.1<br>NM_001033582.1<br>NM_001242874.1<br>NM_002744.4	-2.65[1]	-	-	-	-	-
cAMP-dependent protein kinase catalytic subunit alpha	PRKACA	5566	NM_207518.1<br>NM_002730.3	-	-5.55[1]	-	-	-	-
Tumor necrosis factor receptor superfamily member 1B	TNFRSF1B	7133	NM_001066.2	-	-	2.51[2]	-2.66[2]	-	-
Receptor-interacting serine/threonine-protein kinase 1	RIPK1	8737	NM_003804.3	-	3.45[1]	2.20[2]	-	-	-
Kinectin	KTN1	3895	NM_182926.2	-5.37[1]	-	-	-	-	-

Gene name	Gene symbol	Entrez_ID	RefSeq_ID	Dyslipidemic			Normolipidemic		
				Ratio $t_{4h}:t_0$	Ratio $_{t1}:t_0$	Ratio $t_{12}:t_0$	Ratio $t_{4h}:t_0$	Ratio $_{t1}:t_0$	Ratio $t_{12}:t_0$
**Nuclear factor kappa b pathway [continuation]**									
DNA replication licensing factor MCM5	MCM5	4174	NM_006739.3	-4.07[1]	-	-	-	-	-
Guanine nucleotide-binding protein subunit beta-2-like 1	GNB2L1	10399	NM_006098.4	9.05[3]	6.06[3]	14.73[3]	-	-2.76[2]	-
BAG family molecular chaperone regulator 4	BAG4	9530	NM_004874.2	-	6.19[1]	4.97[1]	-	-	-
Ribosomal protein L4	RPL4	6124	NM_000968.2	-	-3.85[1]	2.91[2]	-	-2.59[2]	-
Ribosomal protein S11	RPS11	6205	NM_001015.3	-	-4.94[2]	2.10[2]	-	-	2.20[3]
Ribosomal protein L30	RPL30	6156	NM_000989.2	-	-	3.34[1]	-	-	2.73[2]
Mitogen-activated protein kinase kinase kinase 3	MAP3K3	4215	NM_203351.1<br>NM_002401.3	-	-3.53[1]	-5.15[1]	-	-	-
Ras GTPase-activating protein-binding protein 2	G3BP2	9908	NM_012297.4<br>NM_203505.2<br>NM_203504.2	-2.93[1]	-3.23[1]	-5.21[1]	-	-	-
14-3-3 protein eta	YWHAH	7533	NM_003405.3	-	-9.33[1]	-	-	-	-
Heat shock protein beta-1	HSPB1	3315	NM_001540.3	-	-2.29[1]	2.29[2]	-	-	-
Mitogen-activated protein kinase kinase kinase 7-interacting protein 1	MAP3K7IP1	10454	NM_006116.2	4.41[1]	16.23[1]	6.79[1]	-	-	-
Mitogen-activated protein kinase kinase kinase 7-interacting protein 1	MAP3K71P2	23118	NM_015093.3	-	-3.91[1]	-9.24[1]	-	-	-
Casein kinase 2, beta polypeptide	CSNK2B	1460	NM_001320.5	-	-	2.81[2]	-2.11[2]	-	-
14-3-3 protein gamma	YWHAG	7532	NM_012479.3	-	-3.29[1]	-2.41[1]	-	-	-
Histone deacetylase 6	HDAC6	10013	NM_006044.2	-	5.46[3]	6.89[3]	-	-	-
SWI/SNF complex subunit SMARCC2	SMARCC2	6601	NM_001130420.1<br>NM_003075.3<br>NM_139067.2	-	-7.44[1]	-2.60[2]	-	-	2.54[1]
DNA-directed RNA polymerases I and III subunit RPAC1	POLR1C	9533	NM_004875.2<br>NM_203290.1	-	-14.84[1]	-5.72[1]	-	-	-
5-azacytidine-induced protein 2	AZI2	64343	NM_022461.3	-	-2.15[1]	-5.29[1]	-	-	-
Mitogen-activated protein kinase kinase kinase 7-interacting protein 2	TXLNA	200081	NM_175852.3	-	5.06[1]	3.45[1]	-	-	-

Gene name	Gene symbol	Entrez_ID	RefSeq_ID	Dyslipidemic			Normolipidemic		
				Ratio $t_{4h} : t_0$	Ratio $_{t1} : t_0$	Ratio $t_{12} : t_0$	Ratio $t_{4h} : t_0$	Ratio $_{t1} : t_0$	Ratio $t_{12} : t_0$
**oxidative stress pathway**									
Glutathione peroxidase 1	GPX1	2876	NM_000581.2<br>NM_201397.1	-	-	-	-	-	-2.48[3]
Extracellular superoxide dismutase [Cu-Zn]	SOD3	6649	NM_003102.2	-	-	4.07[3]	-	-	2.91[3]
Nuclear factor 1 X-type	NFIX	4784	NM_002501.2	-	-3.50[1]	3.04[2]	-2.57[1]	-	-
Superoxide dismutase 2, mitochondrial	SOD2	6648	NM_000636.2	-	-	-	2.68[1]	-	-
Glutathione reductase	GSR	2936	NM_000637.2	-	-	2.38[3]	-	-	-2.54[2]
Transcription factor Sp1	SP1	6667	NM_001251825.1<br>NM_003109.1<br>NM_138473.2	3.91[1]	-	-	-	-	-
Cytochrome b-245 light chain	CYBA	1535	NM_000101.2	-	-3.77[1]	2.61[2]	-	-	-
Heme oxygenase 1	HMOX1	3162	NM_002133.1	-	-8.57[1]	-17.52[1]	-	-	-
Glutathione peroxidase 3	GPX3	2878	NM_002084.3	-	-	-2.15[1]	-	-	-
NAD(P)H dehydrogenase [quinone] 1	NQO1	1728	NM_001025434.1<br>NM_001025433.1<br>NM_000903.2	-	4.66[1]	-	-	-	-
Catalase	CAT	847	NM_001752.2	-	13.15[1]	8.90[1]	-	-	-

- no regulation
[1] slightly significant regulation; P=0.05
[2] significant regulation; P<0.05
[3] highly significant regulation; P<0.01
